# Comprehensive Analysis of *CRIP1* in Patients with Ovarian Cancer, including ceRNA Network, Immune-Infiltration Pattern, and Clinical Benefit

**DOI:** 10.1155/2022/2687867

**Published:** 2022-01-31

**Authors:** Bingli Qi, Shikai Liu, Dantong Liu, Hairong Yao, Ruixue Yan

**Affiliations:** ^1^Department III of Gynecology, Cangzhou Central Hospital, Cangzhou, China; ^2^Department I of Gynecology, Cangzhou Central Hospital, Cangzhou, China

## Abstract

**Background:**

With the development of sequencing technology, an increasing number of biomarkers have been identified in ovarian cancer (OC). However, there have been few comprehensive analyses of *CRIP1* in patients with OC.

**Methods:**

Logistic regression analysis was conducted to analyze the correlations between clinical characteristics and *CRIP1* expression. Kaplan-Meier survival analysis was used to explore the difference in survival in each clinical subgroup. In addition, univariate and multivariate Cox regression analyses were further used to confirm the independent prognostic values of *CRIP1*. We further constructed ceRNA network based on the difference analysis. Subsequently, we used the ssGSEA algorithm to excavate the correlation between *CRIP1* and tumor-infiltrating immune cells. Moreover, the potential biological functions of *CRIP1* were investigated by gene function annotation. Finally, we knocked down *CRIP1* gene for preliminary biological function verification in A2780 and SKOV-3 cell lines.

**Results:**

The overexpression of *CRIP1* was confirmed in The Cancer Genome Atlas (TCGA) cohort, immunohistochemistry, and OC cell lines. *CRIP1* overexpression was correlated with the FIGO stage according to a logistic regression analysis that used the median of *CRIP1* expression as a categorization of the dependent variable. Survival analysis revealed that *CRIP1* was associated with a poor prognosis in most clinical subgroups and acts as an independent prognostic marker in OC patients. In immuno-bioinformatics analysis, *CRIP1* is associated to majority of immune cells. This is noteworthy given that we identified that the ceRNA network based on *CRIP1* may regulate progression in OC. In addition, gene enrichment analysis suggested *CRIP1* may be involved in the JAK-STAT signaling pathway, etc. Finally, we found that knockdown *CRIP1* could inhibit the proliferation of OC cells.

**Conclusion:**

We provided robust evidences that *CRIP1* is an indicator of poor prognosis and a potential target for immunotherapy in patients with OC.

## 1. Introduction

Globally, ovarian cancer (OC) is an important cause of gynecological cancer-related death. Because a large proportion of patients lack specific clinical manifestations in early stage, resulting in 70% of patients being diagnosed at an advanced stage [1], therefore, exploring new diagnostic strategies of OC patients is currently an urgent problem to be addressed.

Cysteine-rich intestinal protein (*CRIP*) is one of the important members of LIM/double zinc-finger protein family [2]. At present, relevant basic research have demonstrated that *CRIP1* is involved in intestinal zinc transport [3] and has a prognostic value in various tumors, such as thyroid cancer [4], acute myeloid leukemia [5], and hypertension-related renal cell carcinoma [6]. Meanwhile, *CRIP1* is overexpressed in immune cells of the epithelium and may play an important role in gut immunity [7]. Furthermore, experimental animal models confirmed that porcine crip1 is activated by Enterococcus faecalis in gastrointestinal epithelial cells [7]. Our study found that this *CRIP1* is overexpressed in all gynecological tumors in pan-cancer analysis. Although one study has discovered the mechanism of *CRIP1* in cervical cancer [8] at present, it has not been studied in depth in other gynecological tumors.

In this study, we deeply investigated the role of *CRIP1* in OC with in vitro assays combined with bioinformatics analysis. We found that *CRIP1* is overexpressed in OC tissues and is associated with multiple immune cells. Meanwhile, we found an important ceRNA axis potentially involved in OC progression. Furthermore, the clinical association and prognosis value of *CRIP1* were explored. Finally, the effect of silencing *CRIP1* on cell proliferation was evaluated in A2780 and SKOV-3 cell lines. In summary, we provided robust evidences that *CRIP1* is an indicator of poor prognosis and a potential target for immunotherapy in patients with OC.

## 2. Materials and Methods

### 2.1. Differential Expression Analysis

We download RNA-sequence data (Level-3 HTseq-FPKM) from the Pan-cancer Project in The Cancer Genome Atlas (TCGA) Database. The expression of *CRIP1* was compared between the normal and tumor tissues after log2-transformation of FPKM raw data. In addition, the RNA-sequence data of 88 normal ovarian epithelial tissues as the same format was downloaded from GTEx database to perform *CRIP1* difference analysis of unpaired (427 OC tissues and 88 adjacent normal tissues). The expression of *CRIP1* protein was excavated from the Human Protein Atlas (HPA) database, including immunohistochemical images and statistical results of staining intensity of different tissues.

### 2.2. Clinical Prognosis and Characteristic Analysis

The clinical data was downloaded from the TCGA database. The clinical characteristics include FIGO stage, age, BMI, histologic grade, FIGO stage, tumor status, lymphatic invasion, anatomic neoplasm subdivison, and tumor residual; survival information include overall survival (OS), disease-specific survival (DSS), and progress-free interval (PFI), as detailed in Table 1. We calculated the median expression of *CRIP1* of OC patients, which is used to select “high-*CRIP1*” and “low-*CRIP1*” groups. Kaplan-Meier survival analysis and log-rank test were used to suggest the survival differences (OS, DSS, and PFI) in two groups. In addition, independent prognostic factors were identified by univariate and multivariate Cox regression analyses. In addition, we used ‘rms' package in R software to plot a nomogram for visualizing the prognosis value of *CRIP1.* The distinction and calibration were evaluated by the ROC curve and calibration curve.

### 2.3. Enrichment Analysis of Differentially Expressed Genes

We divided samples into two groups (high-*CRIP1* and low-*CRIP1*) based on the median expression of *CRIP1*. The differentially expressed genes (DEGs) in *CRIP1*-high samples and *CRIP1*-low samples were screened using ‘limma' package in R software. The thresholds were set to ∣log2(FC) | >2 and *p*.adj < 0.05. Moreover, Gene Ontology (GO) and Kyoto Encyclopedia of Genes and Genomes (KEGG) analyses were performed using related packages. The thresholds were set to *p*.adj < 0.05 and *q* value < 0.2.

### 2.4. Immune-Infiltration Analysis

The immune-infiltration algorithm used in this study was ssGSEA, which was implemented through the ‘GSVA' package in R software. The 24 types of immune cells include aDC, B cells, CD8 T cells, cytotoxic cells, DC, eosinophils, iDC, macrophages, mast cells, neutrophils, NK CD56bright cells, NK CD56dim cells, NK cells, pDC, T cells, T helper cells, Tcm, Tem, Tfh, Tgd, Th1 cells, Th17 cells, Th2 cells, and TReg.

### 2.5. The Construction and Comprehensive Analysis of CRIP1-Related ceRNA Networks

As previously mentioned, we divided samples into two groups (high-*CRIP1* and low-*CRIP1*) based on the median expression of *CRIP1*. The differentially expressed lncRNAs (DELs) in high-*CRIP1* samples and low-*CRIP1* samples were screened using ‘deseq2' package in R software. The thresholds were set to ∣log2(FC) | >2 and *p*.adj < 0.05. We used Starbase (http://starbase.sysu.edu.cn/) to predict target miRNAs of *CRIP1.* Correlation analysis was conducted between all target miRNAs predicted by databases and *CRIP1.* Finally, we only retained miRNAs that was negatively correlated with *CRIP1* and analyzed the prognostic value of the above miRNAs in OC patients. The miRNA (the study was miR-503-5p) involved in the ceRNA network construction were determined by the above methods. At the same time, we also predicted the targeted-lncRNAs of miR-503-5p in the Starbase. Subquently, we constructed the intersection of targeted-lncRNAs and the above DELs as our final lncRNAs involved in the ceRNA network. In addition, we also conducted correlation analysis and survival analysis of the abovementioned lncRNAs. Finally, these genes were combined to construct a lncRNA-miRNA-mRNA *CRIP1* network by Cytoscape software.

### 2.6. Function Detection of CRIP1 in Cell Level

In this study, we used cell culture, transfection, CCK-8, and qRT-PCR as in vitro assays. The Shanghai Cell Institute Country Cell Bank provided the cell lines SKOV-3, A2780, and IOSE80. GenePharma generated and annealed small-interfering RNA (si-RNA-1/2/3) oligos for *CRIP1* and a general negative control. Following the manufacturer's procedure [4], each siRNA duplex was transfected into the cells using Lipofectamine® 2000 (Invitrogen, Carlsbad, CA, USA). A2780 and SKOV-3 cells were planted in a 96-well plate with or without *CRIP1* knockdown. After cell culture for 0, 1, 2, 3, and 4 days, cell viability was determined using the CCK-8 (Dojindo, Tokyo, Japan). In addition, *CRIP1* and *GAPDH* gene primers were as follows: CRIP1-F: 5′-GGAGCGAGATCCCTCCAAAAT-3′, CRIP1-R: 5′-GGCTGTTGTCATACTTCTCATGG-3′ and GAPDH-F: 5′-AATTCGGCACGAGGCATGATCCAA-3′, GAPDH-R: 5′-AGAAGCCCCAGGAAAAGACTGACA-3′. The details of the methods are provided in references [4, 8].

### 2.7. Statistical Analysis

All statistical analyses were performed using the R software (v.3.6.3). Detailed statistical methods about Transcriptome data are covered in the bioinformatics method section. *p* < 0.05 was considered statistically significant.

## 3. Result

### 3.1. *CRIP1* Expression in Pan-Cancer and OC Patients

**Figure 1:**
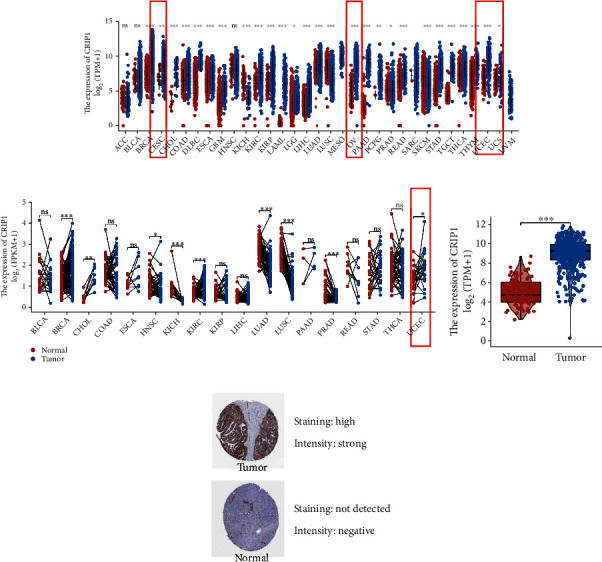
*CRIP1* expression in pan-cancer and OC patients. (a) Differential expression of *CRIP1* in pan-cancer patients, gynecological tumors in the red frame. (b) Differential expression *CRIP1* of paired samples from the TCGA database, gynecological tumors in the red frame. (c) The expression of *CRIP1* was explored by combining normal ovairan tissue samples in GTEx database. (d) Representative Immunohistochemical staining of CRIP1 in the HPA database. ^∗∗^*p* < 0.01, ^∗∗∗^*p* < 0.001.

**Figure 2:**
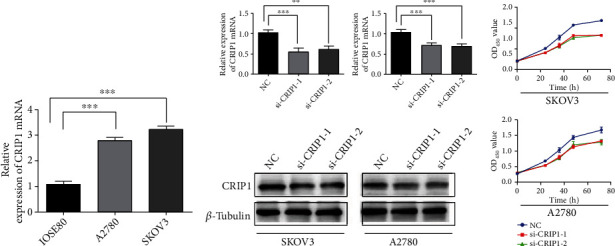
Cell function assays of knockdown *CRIP1.* (a) Relative expression of *CRIP1* mRNA in IOSE80, A2780, and SKOV-3 cell lines. (b) Relative expression of *CRIP1* mRNA and protein in OC cell lines transfected with si-*CRIP1*. (c) CCK8 assays in OC cell lines transfected with si-*CRIP1*. ^∗^*p* < 0.05, ^∗∗^*p* < 0.01, and ^∗∗∗^*p* < 0.001.

Firstly, we identified the expression of *CRIP1* in various cancers and focused on OC. We found that there were significant differences in the expression of *CRIP1* in pan-cancer patients (30/33, *p* < 0.05), as shown in Figures 1(a) and 1(b) (paired samples). Of note, *CRIP1* was upregulated in all gynecological tumors. In addition, we explored expression of *CRIP1* by combining 88 normal ovarian tissue samples in GTEx database, and the same results were found (*p* < 0.001, Figure 1(c)). Meanwhile, representative immunohistochemical staining of *CRIP1* protein in the HPA database revealed a lower expression in normal samples (Figure 1(d)). Not surprisingly, in in vitro assays, the result validated the upregulated *CRIP1* in A2780 and SKOC-3 cell lines (Figure 2(a)).

### 3.2. Identification of the Correlation between *CRIP1* Expression and Clinical Characteristics

**Figure 3:**
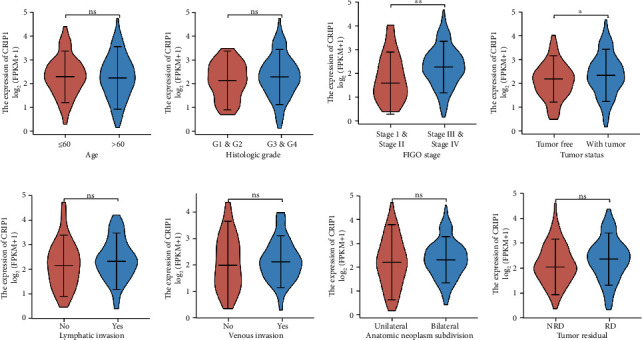
Correlation analysis between *CRIP1* expression and clinical characteristics: (a) age, (b) histologic grade, (c) FIGO stage, (d) tumor status, (e) lymphatic invasion, (f) venous invasion, (g) anatomic neoplasm subdivision, and (h) tumor residual. ^∗^*p* < 0.05, ^∗∗^*p* < 0.01.

Correlation analysis was performed between *CRIP1* expression and corresponding clinical characteristics. As presented in Figure 3, increased expression of *CRIP1* is remarkably related to multiple factors, including FIGO stage (*p* < 0.001, Figure 3(c)) and tumor status (*p* < 0.01, Figure 3(d)). Meanwhile, it should also be noted that the expression of *CRIP1* was not statistically correlated with the following clinical characteristics: age (Figure 3(a)), histologic grade (Figure 3(b)), lymphatic invasion (Figure 3(e)), venous invasion (Figure 3(f)), anatomic neoplasm subdivision (Figure 3(g)), and tumor residual (Figure 3(h)). Furthermore, as shown in Table 2, using the median of *CRIP1* expression as the dependent variable, logistic regression analysis revealed that overexpression of *CRIP1* was significantly linked with FIGO stage (advanced stage vs. early stage, *p* = 0.01).

### 3.3. Prognostic Value and Subgroup Analysis of *CRIP1*

**Figure 4:**
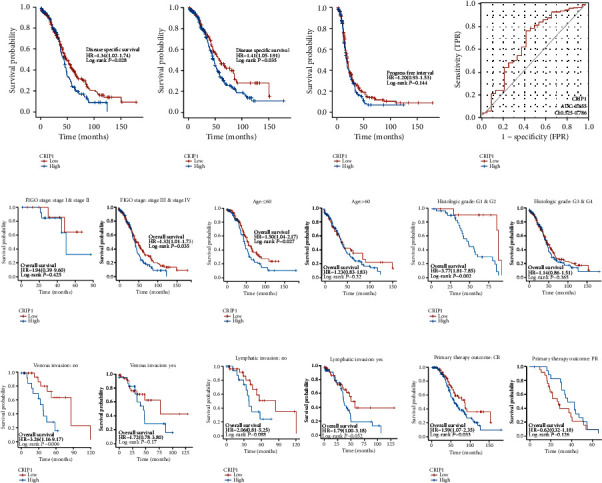
Prognostic value of *CRIP1* in OC Patients. (a) OS: overall survival; (b) DSS: disease-specific survival. (c) PFI: progress-free interval. (d) ROC analysis for OS. Survival analysis of clinical subgroups, including (e) FIGO stage, (f) age, (g) histologic grade, (h) venous invasion, (i) lymphatic invasion, and (j) primary therapy outcome.

To further explore the prognostic value of *CRIP*, we performed survival analysis of clinical subgroup. Firstly, we calculated the median expression of *CRIP1* of OC patients, which is used to select “high-CRIP1” and “low-CRIP1” groups. Kaplan-Meier survival analysis and log-rank test were used to suggest the survival differences (OS, DSS, and PFI) in two groups. The results showed that except for PFS, the survival time of the high-expression group is significantly shorter than the low-expression group (OS, *p* = 0.028, Figure 4(a); DFS, *p* = 0.035, Figure 4(b); PFI, *p* = 0.144, Figure 4(c)). In addition, we also analyzed the prediction value of *CRIP1* in OS, and the ROC curve results showed that *CRIP1* had a good predictive performance (AUC:0.655, Figure 4(d)). In the survival analysis of the clinical subgroups, the survival time of the high-*CRIP1* group was significantly shorter than the low-*CRIP1* group in the advanced group (*p* = 0.035, Figure 4(e)), nonelderly group (*p* = 0.027, Figure 4(f)), G1 & G2 group (*p* = 0.0002, Figure 4(g)), nonvascular invasion group (*p* = 0.006, Figure 4(h)), and primary therapy outcome: CR group (*p* = 0.033, Figure 4(j)). Although there was no significant difference in survival time among the subgroups of lymphatic invasion (no, *p* = 0.088; yes, *p* = 0.052; Figure 4(i)), it is of concern.

### 3.4. Identification of Immune-Infiltration Landscapes Based on *CRIP1*

**Figure 5:**
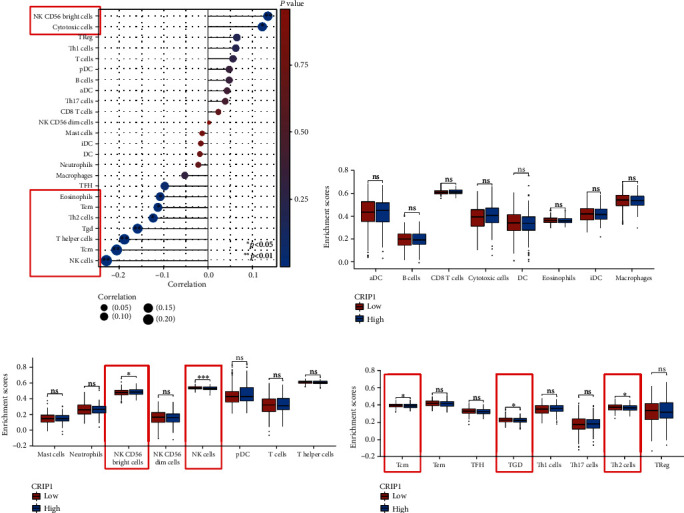
Correlation between *CRIP1* expression and immune infiltration in OC. (a) Person analysis of 24 immune cells and *CRIP1* expression. (c–d) Differential expression analysis of 24 immune cells in patients with different *CRIP1* expressions. ^∗^*p* < 0.05, ^∗∗^*p* < 0.01, and ^∗∗∗^*p* < 0.001.

Especially, infiltrating immune cells are independent predictor of survival in patients with OC. Therefore, we explored the correlation between *CRIP1* and 24 immune cells, as well as the relationship between the expression of *CRIP1* mRNA and immune cells. Based on the median expression value of *CRIP1*, all OC patients were classified into the high- and low-expression groups. It showed that NK CD56bright cells, NK cells, Tcm, Tgd, and Th2 cells differed significantly between groups (Figures 5(b)–5(d), Table 3). Moreover, the results showed that *CRIP1* was negative correlation with only seven immune cells, including eosinophils, Tem, Th2 cells, Tgd, T helper cells, Tcm, and NK cells (Figure 5(a)).

### 3.5. Construction of Nomogram Based on *CRIP1*

**Figure 6:**
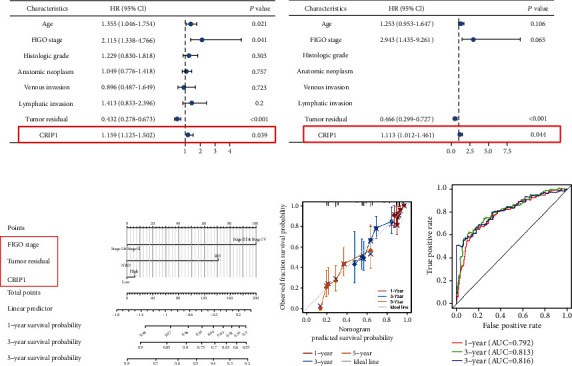
Construction of nomogram based on *CRIP1*. Univariate (a) and multivariate Cox regression analysis (b) based on *CRIP1* and clinicopathologic factors. Nomogram (c), calibration curve (d), and ROC analysis (e) based on tumor residual, FIGO stage, and *CRIP1*.

To investigate the independent prognostic value of *CRIP1* in OC patients, in which univariate Cox analysis revealed that *CRIP1* same age and FIGO stage were high-risk factors, while tumor residual was a low-risk factor (Figure 6(a)). Moreover, further multivariate Cox analysis showed that *CRIP1* and tumor residual were independently associated with OS, which may imply that *CRIP1* may be an independent prognostic predictor for OC patients (Figure 6(b)). Meanwhile, considering the clinical value of FIGO stage, we combined FIGO stage and significance factors in multivariate analysis to construct a visual prognostic model (Figure 6(c)). The ROC curve and calibration curve also showed that the model had better predictive value (Figures 6(d) and 6(e)).

### 3.6. The Construction and Comprehensive Analysis of CRIP1-Related ceRNA Networks

**Figure 7:**
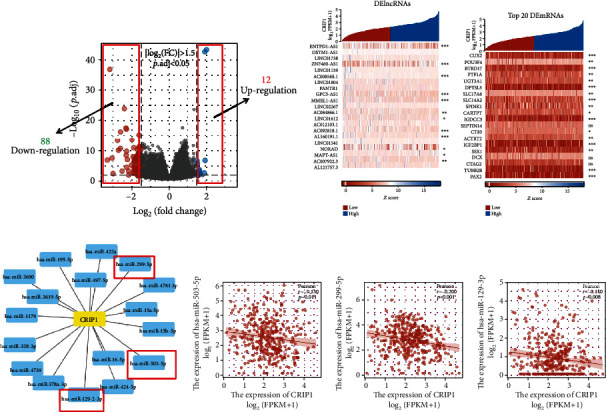
Identification of targeted-miRNAs and screening of DElncRNAs. (a) Volcanic plot of differential gene expression. (b) The heatmap of DElncRNAs in OC patients. (c) The heatmap of DEGs in OC patients. (d) A network of *CRIP1* and target miRNAs. The Pearson correlation analysis of miR-503-5p (e), miR-299-5p (f), and miR-129-2-3p (g).

We divided samples into two groups (high-*CRIP1* and low-*CRIP1*) based on the median expression of *CRIP1*. The DELs and DEGs in two groups were screened using the thresholds set to ∣log2(FC) | >2 and *p*.adj < 0.05. Finally, we screened out 100 differential genes, of which 88 were downregulated and 12 upregulated (Figure 7(a)). The gene heatmaps showed the DELs (Figure 7(b)) and the top 20 DEGs (Figure 7(c)).

**Figure 8:**
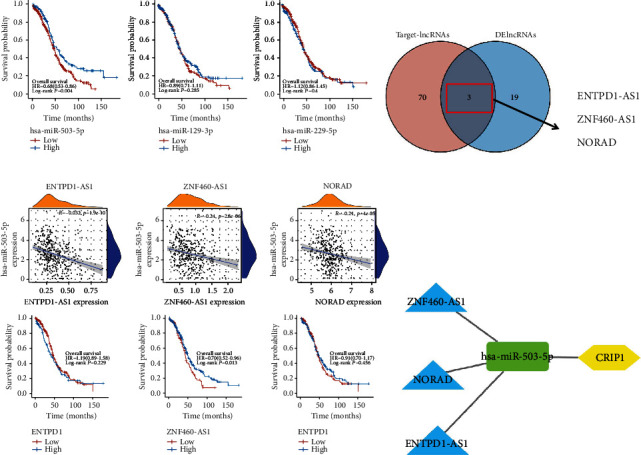
Construction of ceRNA network based on *CRIP1*. (a) Survival analysis of target miRNAs. (b) A Venn plot for target lncRNAs and DElncRNAs. The Pearson correlation analysis and survival analysis of ENTPD1-AS1 (c), ZNF460-AS1 (d), and NORAD (e). (f) A ceRNA network based on *CRIP1.*

To build the ceRNA network, we used Starbase to predict target miRNAs of *CRIP1* (Figure 7(d)), subsequently, correlation analysis was conducted between all target miRNAs predicted by databases and *CRIP1*. Only three target miRNAs were negatively correlated with *CRIP1* in OC, including miR-503-5p (*r* = −0.13, *p* = 0.11, Figure 7(e)), miR-299-5p (*r* = −0.2, *p* < 0.001, Figure 7(f)), and miR-129-2-3p (*r* = −0.14, *p* = 0.008, Figure 7(g)). Meanwhile, we analyzed the prognostic value of the above miRNAs in OC patients. The miR-503-5p with prognostic value was included in the construction of the ceRNA network (*p* = 0.004, HR = 0.68, Figure 8(a)). Moreover, we also predicted the targeted lncRNAs of miR-503-5p in Starbase. In the following, we constructed the intersection of targeted lncRNAs and the above DELs as our final lncRNAs involved in the ceRNA network (Figure 8(b)). Similar to miRNA treatment, we also performed correlation analysis and survival analysis for above lncRNAs in intersection (*ENTPD1-AS1*, *ZNF460-AS1*, and *NORAD*). All the three lncRNAs were negatively correlated with miR-503-5p (Figures 8(c) and 8(d)), and only *ZNF460-AS1* showed statistical difference in the survival analysis (*p* = 0.013). Finally, these genes were combined to construct a *ZNF460-AS1/NORAD/ENTPD1-AS1*-miR-503-5p-*CRIP1* network (Figure 8(f)).

### 3.7. Conjecture of the Potential Mechanisms of *CRIP1*

**Figure 9:**
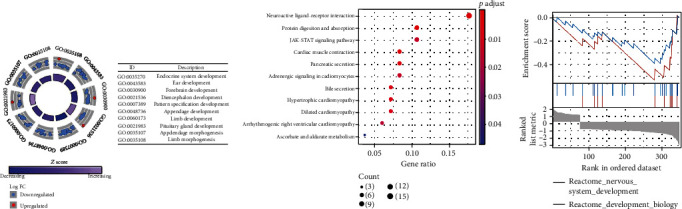
Gene enrichment analysis of DEGs. (a) GO enrichment analysis. (b) KEGG enrichment analysis. (c) GSEA enrichment analysis.

In order to explore the potential mechanism of C*RIP1* involvement, we performed gene enrichment analysis on all the above DEGs. In the results of GO enrichment analysis of DEGs, the genes were mainly enriched in endocrine system development, hormone binding, and forebrain development, etc. (Figure 9(a)). In the results of KEGG pathway enrichment analysis of DEGs, the genes were mainly enriched in protein digestion and absorption, neuroactive ligand-receptor interaction, and cardiac muscle contraction, etc. (Figure 9(b)). In addition, we also conducted GSEA enrichment analysis on the above genes, and the results showed that these genes may be related to the process of nervous system development (Figure 9(c)).

### 3.8. *CRIP1* Expression Analyzed In Vitro

To further validate the above bioinformatics results, we detected the expression level of *CRIP1* mRNA in OC cell lines. The results showed that the expression of *CRIP1* was upregulated in OC cancer cell lines (A2780 and SKOV-3) compared to IOSE80, as shown in Figure 2(a). In addition, si-*CRIP1* and si-NC were transfected in A2780 and SKOV-3 cells, respectively, and qRT-PCR and western blot were used to detect the protein expression of *CRIP1*. It was found that *CRIP1* expression was downregulated in OC cell lines with transfection (Figure 2(b)). Similarly, CCK-8 assays showed that OC cell proliferation was inhibited after transfection with *CRIP1* (Figure 2(c)).

## 4. Discussion

With the rapid development of second-generation sequencing and bioinformatics, it is easier to obtain differentially expressed genes in OC [9]. It provides a broader idea, better conditions, and new perspective for the pathogenesis of OC. This study focused on the prognostic value and potential mechanism of *CRIP1* in OC. Although *CRIP1* can promote or inhibit the development of different tumors under different conditions, it has not been discussed in detail in OC.

The correlation between *CRIP1* expression and clinical variables was investigated using logistic regression analysis and clinical correlation analysis. We also performed Kaplan-Meier and Cox regression analyses to see if there was an association between *CRIP1* expression and survival outcomes. We also analyzed the relationship between *CRIP1* expression and immune-infiltrating cells. The possible mechanism of *CRIP1* was then investigated using GO and KEGG. Finally, in OC cell lines, the ceRNA regulation network based on *CRIP1* was built and confirmed. To our knowledge, no study has been done on *CRIP1* expression in OC and its possible predictive significance. *CRIP1* has been demonstrated in a few studies to operate as an oncogene. Zhang et al. [8] found that overexpression of *CRIP1* in cervical cancer was associated with shorter survival time. Our data also support the carcinogenic effect of *CRIP1*. On the contrary, a few studies have shown that CRIP1 may have an antitumor effect. Baumhoer et al. [10] found that *CRIP1* was associated with good prognosis and less metastasis in patients with osteosarcoma.

In conclusion, based on public sequencing and clinical data, we constructed ceRNA regulatory network based on *CRIP1* expression. We further explored the role of *CRIP1* in ovarian cancer by qRT-PCR and western blot in the OC cell line. This study may provide a new understanding of the pathogenesis of OC and reveal potential therapeutic targets.

## 5. Conclusions

We provided robust evidences that *CRIP1* is an indicator of poor prognosis and a potential target for immunotherapy in patients with OC.

## Figures and Tables

**Table 1:** Clinical characteristics of OC patients in the TCGA database.

Characteristic	Overall
*n*	379
Age, *n* (%)	
≤60	208 (54.9%)
>60	171 (45.1%)
FIGO stage, *n* (%)	
Stage I	1 (0.3%)
Stage II	23 (6.1%)
Stage III	295 (78.5%)
Stage IV	57 (15.2%)
Histologic grade, *n* (%)	
G1	1 (0.3%)
G2	45 (12.2%)
G3	322 (87.3%)
G4	1 (0.3%)
Anatomic neoplasm, *n* (%)	
Unilateral	102 (28.6%)
Bilateral	255 (71.4%)
Venous invasion, *n* (%)	
No	41 (39%)
Yes	64 (61%)
Lymphatic invasion, *n* (%)	
No	48 (32.2%)
Yes	101 (67.8%)
Tumor residual, *n* (%)	
NRD	67 (20%)
RD	268 (80%)
Tumor status, *n* (%)	
Tumor free	72 (21.4%)
With tumor	265 (78.6%)

**Table 2:** Logistic regression analysis between *CRIP1* expression and clinical characteristics.

Characteristics	Odds ratio (OR)	*p* value
Age (>60 vs. ≤60)	0.954 (0.758-1.200)	0.689
FIGO stage (stage III & stage IV vs. stage I & stage II)	1.909 (1.175-3.176)	0.010
Primary therapy outcome (PD vs. PR & CR)	1.037 (0.663-1.619)	0.872
Histologic grade (G3 & G4 vs. G1 & G2)	1.276 (0.894-1.834)	0.182
Venous invasion (yes vs. no)	1.101 (0.703-1.742)	0.676
Lymphatic invasion (yes vs. no)	1.262 (0.859-1.881)	0.242
Anatomic neoplasm (bilateral vs. unilateral)	1.200 (0.926-1.564)	0.171
Tumor residual (NRD vs. RD)	0.749 (0.544-1.024)	0.072

**Table 3:** Correlation analysis between *CRIP1* and immune cells.

Genes	Immune cells	Coefficient	*p* value
CRIP1	aDC	0.043	0.399
CRIP1	B cells	0.048	0.350
CRIP1	CD8 T cells	0.024	0.646
CRIP1	Cytotoxic cells	0.124	0.016
CRIP1	DC	-0.018	0.726
CRIP1	Eosinophils	-0.108	0.036
CRIP1	iDC	-0.016	0.758
CRIP1	Macrophages	-0.052	0.311
CRIP1	Mast cells	-0.013	0.803
CRIP1	Neutrophils	-0.021	0.677
CRIP1	NK CD56bright cells	0.136	0.008
CRIP1	NK CD56dim cells	0.002	0.962
CRIP1	NK cells	-0.229	<0.001
CRIP1	pDC	0.048	0.349
CRIP1	T cells	0.057	0.270
CRIP1	T helper cells	-0.189	<0.001
CRIP1	Tcm	-0.206	<0.001
CRIP1	Tem	-0.113	0.028
CRIP1	TFH	-0.097	0.059
CRIP1	Tgd	-0.159	0.002
CRIP1	Th1 cells	0.063	0.221
CRIP1	Th17 cells	0.039	0.444
CRIP1	Th2 cells	-0.124	0.016
CRIP1	TReg	0.065	0.204

## Data Availability

The following information was supplied regarding data availability. Data is available at the TCGA (https://portal.gdc.cancer.gov/).

## References

[1] Lheureux S., Braunstein M., Oza A. M. (2019). Epithelial ovarian cancer: evolution of management in the era of precision medicine. *CA: a Cancer Journal for Clinicians*.

[2] O'Dell B. L. (1992). Cysteine-rich intestinal protein (CRIP): a new intestinal zinc transport protein. *Nutrition Reviews*.

[3] He G., Zhu H., Yao Y. (2019). Cysteine-rich intestinal protein 1 silencing alleviates the migration and invasive capability enhancement induced by excessive zinc supplementation in colorectal cancer cells. *American Journal of Translational Research*.

[4] Li H., Zhao L. H., Zhang Z. H. (2018). The impact of cysteine-rich intestinal protein 1 (CRIP1) on thyroid carcinoma. *Cellular Physiology and Biochemistry*.

[5] Ma B. B., Li Y., Wen J., Zhao Y. (2020). m6A RNA methylation regulators contribute to malignant development and have a clinical prognostic effect on cervical cancer. *American Journal of Translational Research*.

[6] Huang W., Wu K., Wu R., Chen Z., Zhai W., Zheng J. (2020). Bioinformatic gene analysis for possible biomarkers and therapeutic targets of hypertension-related renal cell carcinoma. *Translational Andrology and Urology*.

[7] Cai H., Chen J., Liu J. (2017). CRIP1, a novel immune-related protein, activated by *Enterococcus faecalis* in porcine gastrointestinal epithelial cells. *Gene*.

[8] Zhang L. Z., Huang L. Y., Huang A. L., Liu J. X., Yang F. (2018). CRIP1 promotes cell migration, invasion and epithelial-mesenchymal transition of cervical cancer by activating the Wnt/*β*‑catenin signaling pathway. *Life Sciences*.

[9] Tariq H., Gul A., Khadim T. (2021). Next generation sequencing-based germline panel testing for breast and ovarian cancers in Pakistan. *Asian Pacific Journal of Cancer Prevention*.

[10] Baumhoer D., Elsner M., Smida J. (2011). CRIP1 expression is correlated with a favorable outcome and less metastases in osteosarcoma patients. *Oncotarget*.

